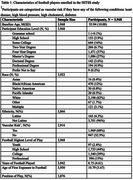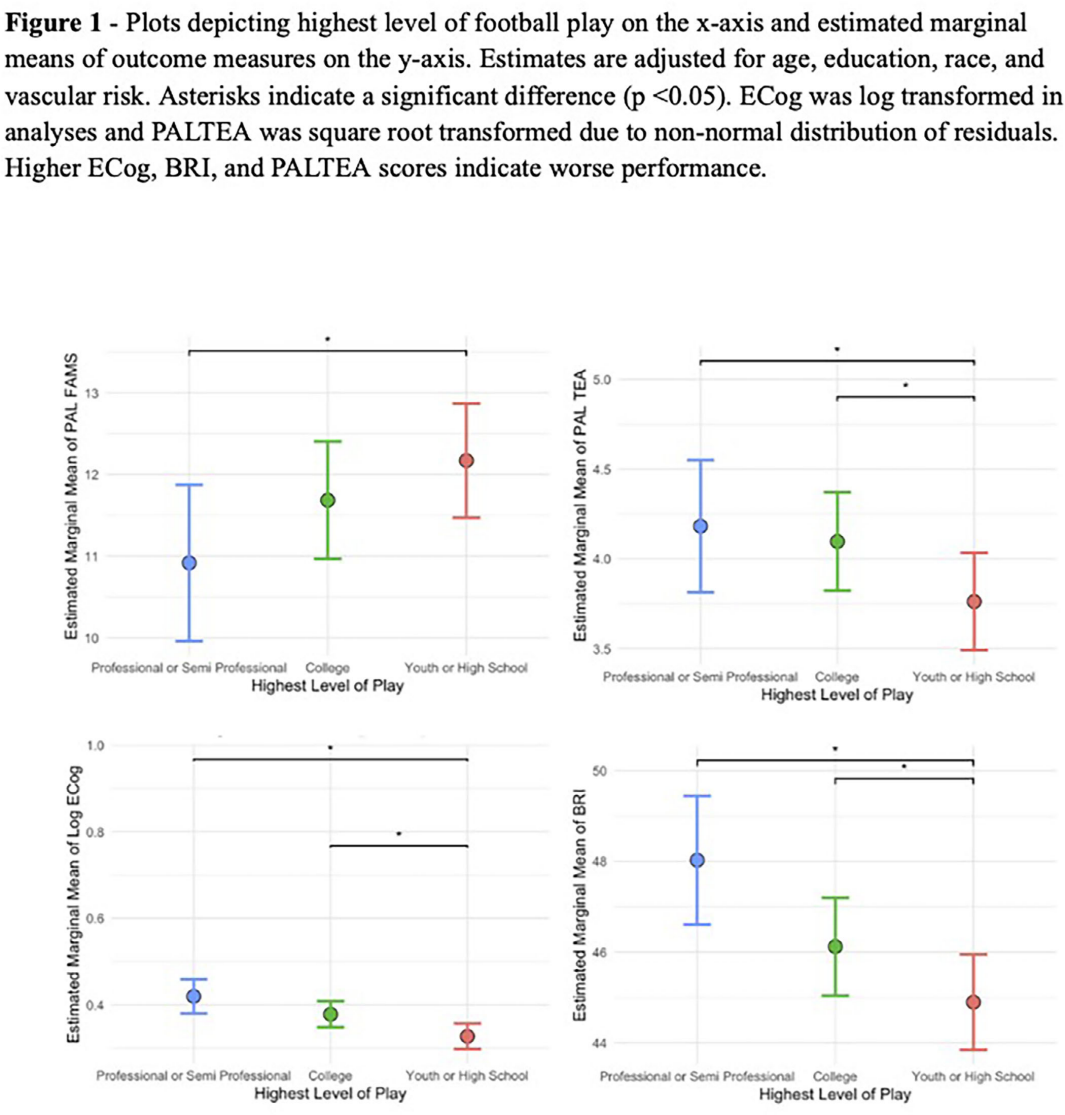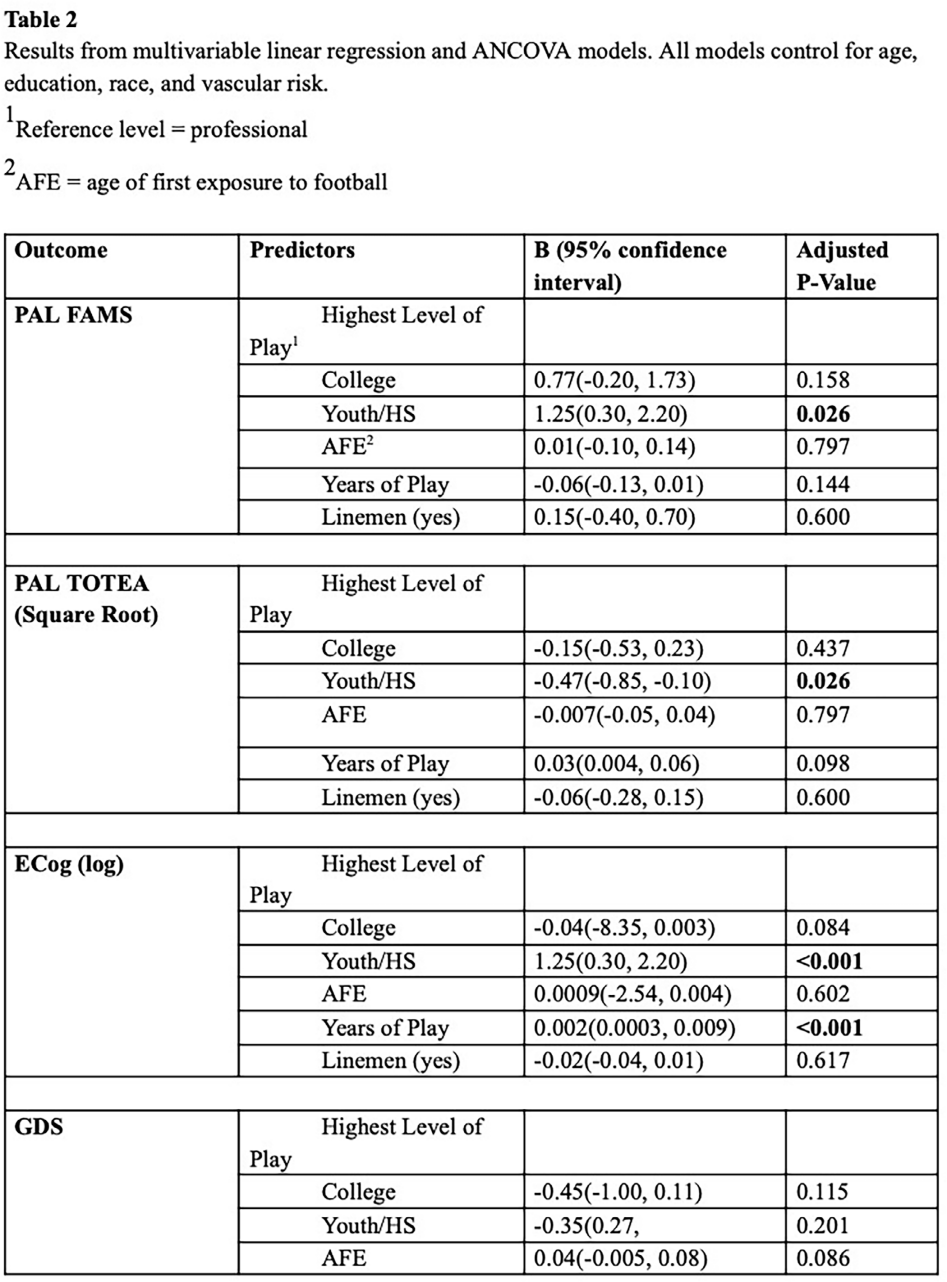# Later‐Life Cognitive and Neuropsychiatric Function in Former American Football Players: Results from the Head Impact & Trauma Surveillance Study

**DOI:** 10.1002/alz70861_108849

**Published:** 2025-12-23

**Authors:** Grace H Badlam, Anna Aaronson, Shania C. Mulayi, Kelsey Goostrey, Yorghos Tripodis, William S Cole‐French, Matthew Roebuck, Brittany N. Pine, Joseph N. Palmisano, Brett Martin, Douglas I Katz, Scott R. Mackin, Michael D McClean, Ann C. McKee, Jennifer Weuve, Jesse Mez, Michael W Weiner, Rachel L. Nosheny, Robert A. Stern, Michael L Alosco

**Affiliations:** ^1^ Boston University, Boston, MA USA; ^2^ Boston University CTE and Alzheimer's Disease Research Center, Boston, MA USA; ^3^ Boston University Chobanian & Avedisan School of Medicine, Boston, MA USA; ^4^ Boston University Chronic Traumatic Encephalopathy, Chobanian & Avedisian School of Medicine, Boston, MA USA; ^5^ Boston University Chronic Traumatic Encephalopathy Center, Boston, MA USA; ^6^ Boston University Alzheimer’s Disease Research Center, Boston, MA USA; ^7^ Boston University Chobanian & Avedisian School of Medicine, Boston, MA USA; ^8^ University of California, San Francisco Department of Psychiatry, San Francisco, CA USA; ^9^ Boston University School of Public Health, Boston, MA USA; ^10^ Boston University Chronic Traumatic Encephalopathy Center, Boston University Chobanian & Avedisian School of Medicine, Boston, MA USA; ^11^ San Francisco Veterans Administration Medical Center (SFVAMC), San Francisco, CA, CA USA; ^12^ University of California, San Francisco, San Francisco, CA USA

## Abstract

**Background:**

Repetitive head impacts (RHI) from contact and collision sports have been associated with later‐life cognitive and neuropsychiatric changes. However, findings have been limited by small sample sizes of college and/or professional American football players. Here, we examined associations between football play across four levels (youth, high school, college, professional) and cognitive and neuropsychiatric outcomes in a large sample.

**Method:**

The sample included 3,968 male American football players enrolled in the Head Impact & Trauma Surveillance Study (HITSS), a national online study of former American football and soccer players, aged 40+ years. Participants complete computerized cognitive tests, and self‐report cognitive, mood, sports history, and behavioral questionnaires. Cognitive outcomes for this study included the Cambridge Neuropsychological Test Automated Battery (CANTAB) Paired Associates Learning First Attempt Memory Score (PALFAMS) and PAL Total Errors adjusted (PALTEA), BRIEF‐A Meta‐Cognition Index (MI), and the Everyday Cognition Scale (ECog). Neuropsychiatric outcomes included the BRIEF‐A Behavioral Regulation Index (BRI) and the Geriatric Depression Scale‐15 (GDS‐15). For each outcome, multivariable linear regressions examined associations for continuous proxies of RHI (total years of football play, age of first exposure); ANCOVAs were used for categorical predictors (linemen vs non‐linemen; highest level of football play). Models controlled for age, education, race, and vascular risk. *p* ‐values were false discovery rate adjusted.

**Result:**

Table 1 provides sample characteristics. Among the participants (mean age 55.95, 12% Black), 46% played at the youth/high school level (youth combined with high school due to small sample size), 39% played at the college and 15% at the professional level. Utilizing omnibus test, the ANCOVA models demonstrated a significant effect for highest level of play on the PALTEA, PALFAMs, ECog, and BRI (padj<0.05), meaning professional players had worse scores compared to college and high school/youth; college players had worse scores than high school/youth players (Figure 1). Multivariable linear regressions revealed significant associations between total years of football play and higher (worse) BRI and ECog scores (Table 2).

**Conclusion:**

In this sample of 3,968 former American football players, findings support a dose‐response relationship between football play and cognitive and neuropsychiatric function.